# Cytogenetic data on six leafcutter ants of the genus *Acromyrmex* Mayr, 1865 (Hymenoptera, Formicidae, Myrmicinae): insights into chromosome evolution and taxonomic implications

**DOI:** 10.3897/CompCytogen.v10i2.7612

**Published:** 2016-05-11

**Authors:** Luísa Antônia Campos Barros, Hilton Jeferson Alves Cardoso de Aguiar, Cléa dos Santos Ferreira Mariano, Vanderly Andrade-Souza, Marco Antonio Costa, Jacques Hubert Charles Delabie, Silvia das Graças Pompolo

**Affiliations:** 1 Laboratório de Citogenética de Insetos, Departamento de Biologia Geral, Universidade Federal de Viçosa; Viçosa - MG, 36570-000, Brazil; 2 Universidade Federal do Amapá; Campus Binacional, Oiapoque - AP, 68980-000, Brazil; 3 Laboratório de Mirmecologia, CEPEC/CEPLAC; Itabuna - BA, CP 7, 45600-000, Brazil; 4 Departamento de Ciências Biológicas, Universidade Estadual de Santa Cruz; Ilhéus - BA, 45650-000, Brazil; 5 Instituto Nacional de Pesquisas da Amazônia; Manaus - AM, 69067-375, Brazil; 6 Departamento de Ciências Agrárias e Ambientais, Universidade Estadual de Santa Cruz; Ilhéus - BA, 45650-000, Brazil

**Keywords:** Chromosome evolution, karyotype, fungus-growing ants, biodiversity, heterochromatin, FISH

## Abstract

Cytogenetic data for the genus *Acromyrmex* Mayr, 1865 are available, to date, for a few species from Brazil and Uruguay, which have uniform chromosome numbers (2n = 38). The recent cytogenetic data of *Acromyrmex striatus* (Roger, 1863), including its banding patterns, showed a distinct karyotype (2n = 22), similar to earlier studied *Atta* Fabricius, 1804 species. Karyological data are still scarce for the leafcutter ants and many gaps are still present for a proper understanding of this group. Therefore, this study aimed at increasing cytogenetic knowledge of the genus through the characterization of other six species: *Acromyrmex balzani* (Emery, 1890), *Acromyrmex coronatus* Fabricius, 1804, *Acromyrmex disciger* (Mayr, 1887), *Acromyrmex echinatior* (Forel, 1899), *Acromyrmex niger* (Smith, 1858) and *Acromyrmex rugosus* (Smith, 1858), all of which were collected in Minas Gerais – Brazil, except for *Acromyrmex echinatior* which was collected in Barro Colorado – Panama. The number and morphology of the chromosomes were studied and the following banding techniques were applied: C-banding, fluorochromes CMA_3_ and DAPI, as well as the detection of 45S rDNA using FISH technique. All the six species had the same chromosome number observed for already studied species, i.e. 2n = 38. *Acromyrmex balzani* had a different karyotype compared with other species mainly due to the first metacentric pair. The heterochromatin distribution also showed interspecific variation. Nevertheless, all the studied species had a pair of bands in the short arm of the first subtelocentric pair. The fluorochrome CMA_3_ visualized bands in the short arm of the first subtelocentric pair for all the six species, while *Acromyrmex rugosus* and *Acromyrmex niger* also demonstrated in the other chromosomes. The AT-rich regions with differential staining using DAPI were not observed. 45S ribosomal genes were identified by FISH in the short arm of the first subtelocentric pair in *Acromyrmex coronatus*, *Acromyrmex disciger* and *Acromyrmex niger*. The uniform chromosome number in the genus *Acromyrmex* (2n = 38) suggests that *Acromyrmex striatus* (2n = 22) should be transferred to a new genus. Other aspects of the chromosome evolution in ants are also discussed.

## Introduction

Fungus-growing ants belong to the *Atta*-genus group (Ward et al. 2015) corresponding to the tribe Attini in the traditional sense. Leafcutter ants comprise a particular group of fungus-growing ants which are referred to as dominant herbivores of the Neotropics (Hölldobler and Wilson 1990). They include the genera *Acromyrmex* Mayr, 1865 and *Atta* Fabricius, 1804 and are exclusively found in the New World, primarily in the Neotropical region (Mayhé-Nunes and Jaffé 1998) and are considered the most derived group of ants arising about 8-12 million years ago (Schultz and Brady 2008, Mehdiabadi and Schultz 2010).

The genus *Acromyrmex* contains 33 described species (or more than 60 taxa if all subspecies and variations are included) (Bolton 2014, Rabeling et al. 2015). They are distributed from California (USA) to Patagonia (Argentina), excluding Chile. Most Brazilian species are widely distributed, although some of them have more restricted distribution (Gonçalves 1961, Mayhé-Nunes 1991, Delabie et al. 2011).

The genus *Acromyrmex* has been subdivided into two subgenera, *Acromyrmex* and *Moellerius* Forel, 1893 (Emery 1913), based on morphological traits. A phylogenetic study based on the morphological traits of this genus showed that the two subgenera formed distinct groups, of which *Moellerius* was considered the most derived (Mayhé-Nunes 1991). However, recent phylogenetic molecular studies of the genus *Acromyrmex*, including five species of the subgenus *Moellerius*, subdivided *Acromyrmex* species into distinct clusters (Cristiano et al. 2013). Only two of them, *Acromyrmex balzani* (Emery, 1890) and *Acromyrmex landolti* (Forel, 1885), were placed in the same group, suggesting that *Acromyrmex* and *Moellerius* could not be monophyletic (Cristiano et al. 2013); similar results were observed by Sumner et al. (2004). These data suggest that the two subgenera *Acromyrmex* and *Moellerius* do not represent natural groups.

Leafcutter ants are one of the most studied groups of fungus-growing ants (Mayhé-Nunes 1991), both in terms of biology and geographic distribution. Their status as agricultural pests has contributed to their knowledge, although taxonomic limits of different species are sometimes unclear (Delabie et al. 2011, Bacci et al. 2009). Under these circumstances, the so-called “integrative taxonomy” can produce more consistent results by complementing data obtained by different techniques (Schlick-Steiner et al. 2010). Nowadays, ant cytogenetics is a rapidly developing research field (Delabie et al. 2012). Cytogenetic data on fungus-growing ants with information for at least the chromosome number and morphology are available at present for 38 taxa (reviewed in Barros et al. 2011, Cristiano et al. 2013, Barros et al. 2013, 2014a, 2014b, Cardoso et al. 2014, Barros et al. 2015), corresponding to about 10% of described species (Brandão et al. 2011). In some ant genera, e.g. in *Mycetarotes* Emery, 1913 and *Cyphomyrmex* Mayr, 1862, chromosome numbers are variable at the species level (reviewed in Barros et al. 2011). However, species within the genera *Atta* (Barros et al. 2014a, Fadini and Pompolo 1996, Murakami et al. 1998, Barros et al. 2015) and *Acromyrmex* (Fadini and Pompolo 1996, Goñi et al. 1983) have the same chromosome numbers, 2n = 22 and 2n = 38, respectively, and similar chromosome morphology.

Cytogenetic data on the leafcutter ants are scarce. Namely, these data are available for five *Atta* species (Barros et al. 2014a, Fadini and Pompolo 1996, Murakami et al. 1998, Barros et al. 2015). In these species, 2n = 22 and a karyotypic formula of 2n = 18m+2sm+2st were found. Similar banding patterns were also observed in different species (Barros et al. 2014a, Murakami et al. 1998, Barros et al. 2015) which belonged to the three of four species groups defined on the basis of molecular data (Bacci et al. 2009). Cytogenetic data on *Acromyrmex* are also restricted but available for some taxa collected in Brazil: Acromyrmex (Acromyrmex) crassispinus (Forel, 1909); Acromyrmex (Acromyrmex) subterraneus molestans Santschi, 1925; Acromyrmex (Acromyrmex) subterraneus subterraneus Forel, 1893 (Fadini and Pompolo 1996); and in Uruguay: Acromyrmex (Acromyrmex) ambiguus Emery, 1888; Acromyrmex (Acromyrmex) hispidus Santschi, 1925; and Acromyrmex (Moellerius) heyeri (Forel, 1899) (Goñi et al. 1983). All the species had the same chromosome number, 2n = 38. However, Acromyrmex (Moellerius) striatus (Roger, 1863) has recently shown 2n = 22, with a karyotypic formula of 2n = 20m+2sm (Cristiano et al. 2013), the same chromosome number found in all *Atta* species studied to date (Barros et al. 2014a, Fadini and Pompolo 1996, Murakami et al. 1998, Barros et al. 2015). Since *Acromyrmex striatus* belongs to the well-supported clade which is quite distinct from other members of the genus *Acromyrmex*, it is suggested that this species is a sister group of all other leafcutter ants, which split before the divergence between *Acromyrmex* and *Atta* (Cristiano et al. 2013). Despite the same chromosome number, karyotypes of *Acromyrmex striatus* and *Atta* species differ in morphology of two chromosome pairs as well as in their banding patterns (Cristiano et al. 2013, Barros et al. 2014a). The aim of the present study is therefore to describe chromosome sets of six species of the genus *Acromyrmex* to update our knowledge of karyotype evolution of leafcutter ants and Neotropical Formicidae in general.

## Material and methods

Six cytogenetically studied *Acromyrmex* species were collected between August 2008 and March 2010 in the state of Minas Gerais – Brazil, except for *Acromyrmex echinatior* (Forel, 1899) which was collected in Panama (Table 1). Metaphases were obtained according to Imai et al. (1988) using larval ganglia or testes of freshly defecated larvae. To study chromosome morphology, metaphases were analyzed using conventional 4% Giemsa staining. The karyotypes were composed by arranging chromosomes according to their size and chromosome arm ratio (r) (Levan et al. 1964). Ten best metaphases per species with a similar degree of condensation were measured. Karyotypes were composed using the Corel Photopaint X3® software. The colonies and individuals analyzed are listed in Table 1. For the banding techniques, 4 to 10 individuals per species were used: C-banding for heterochromatin detection was performed according to Sumner (1972) with minor adaptations suggested by Barros et al. (2013); sequential fluorochrome staining with CMA_3_/DA/DAPI (Schweizer 1980) was done to reveal specific GC- and AT-rich regions. To detect nucleolus organizer regions (NORs) in three species, 2 to 4 individuals of each species were studied using fluorescence *in situ* hybridization (FISH) with the 45S rDNA probe isolated from *Arabidopsis thaliana* (Moscone et al. 1996). The metaphases were observed and photographed using an Olympus® BX 60 microscope attached to a Q Color 3 Olympus® image capture system. For fluorochrome analysis, filters WB (450-480 nm) and WU (330-385 nm) were used for studying CMA_3_ and DAPI staining, respectively, as well as Leica microscope DMRA2 filter Y3 (545/30 nm), attached to a D Leica IM50 Version 5 Release 190 software was used for FISH analysis.

**Table 1.** *Acromyrmex* spp. cytogenetically studied in this paper. Locality, sample size (number of colonies/individuals stained with Giemsa), diploid (2n) and haploid (n) chromosome number and karyotypic formula.

*Acromyrmex* species	Locality (coordinates)	Colony – Individuals	2n (n)	Karyotypic formula
Acromyrmex (Moellerius) balzani (Emery, 1890)	Viçosa – MG – Brasil (20°45'S; 42°51'W)	3 – 12	38	2n = 12m + 10sm + 14st + 2a
Acromyrmex (Moellerius) balzani (Emery, 1890)	Paraopeba – MG – Brazil (19° 17'S; 44° 29'W)	2 – 15	38	2n = 12m + 10sm + 14st + 2a
Acromyrmex (Acromyrmex) coronatus Fabricius, 1804	São Tiago – MG – Brazil (20°54'S; 44°30'W)	1 – 10	38 (19)	2n = 12m + 8sm + 16st + 2a
Acromyrmex (Acromyrmex) coronatus Fabricius, 1804	Paraopeba – MG – Brazil (19°17'S; 44°29'W)	5 – 20	38	2n = 12m + 8sm + 16st + 2a
Acromyrmex (Acromyrmex) disciger (Mayr, 1887)	Santos Dumont – MG – Brazil (21°27'S; 43°32'W)	2 – 15	38	2n = 10m + 12sm + 14st + 2a
Acromyrmex (Acromyrmex) niger (Smith, F. 1858)	Viçosa – MG – Brazil (20°45'S; 42°51'W)	3 – 21	38	2n = 12m + 14sm + 10st + 2a
Acromyrmex (Acromyrmex) rugosus (Smith, F. 1858)	Florestal – MG – Brazil (19°52'S; 44°24'W)	1 – 6	38	2n = 16m + 12sm + 8st + 2a
Acromyrmex (Acromyrmex) rugosus (Smith, F. 1858)	Paraopeba – MG – Brazil (19° 17'S; 44° 29'W)	5 – 22	38	2n = 16m + 12sm + 8st + 2a
Acromyrmex (Acromyrmex) echinatior (Forel, 1899)	Barro Colorado – Panama (9°9'N; 79°50'W)	2 – 10	38	2n = 8m + 6sm + 14st + 10a

Adult ant specimens were identified by J.H.C. Delabie and deposited in the ant collection at the Laboratório de Mirmecologia do Centro de Pesquisas do Cacau (CPDC/Brazil).

## Results

**Figure 1.**
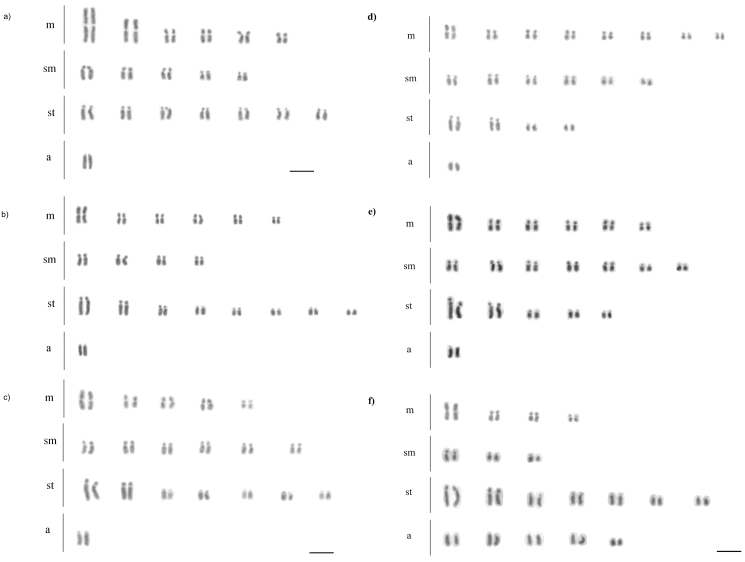
Karyotype of *Acromyrmex* species. **a** *Acromyrmex balzani* **b** *Acromyrmex coronatus* **c** *Acromyrmex disciger* **d** *Acromyrmex rugosus* **e** *Acromyrmex niger* **f** *Acromyrmex echinatior*. All species have 2n = 38. Bar = 5 µm.

All studied species had the same diploid chromosome number, 2n = 38 (Table 1, Fig. 1); males of *Acromyrmex coronatus* Fabricius, 1804 showed n = 19 (Table 1).

Chromosome measurements revealed morphological differences between similar karyotypes (Table 1). Several chromosome pairs were easily recognizable among different species, i.e. the first metacentric pair, the two largest subtelocentric pairs and the largest (or unique in some species) acrocentric pair (Fig. 1). In the karyotype of *Acromyrmex balzani*, the first metacentric pair was larger than in any other studied species (Fig. 1). The size of the first metacentric and the first subtelocentric chromosome pairs were similar in all species (Fig. 1b–f), again except for *Acromyrmex balzani* (Fig. 1a). No geographical intraspecific differences between the karyotypes were found (Table 1).

**Figure 2.**
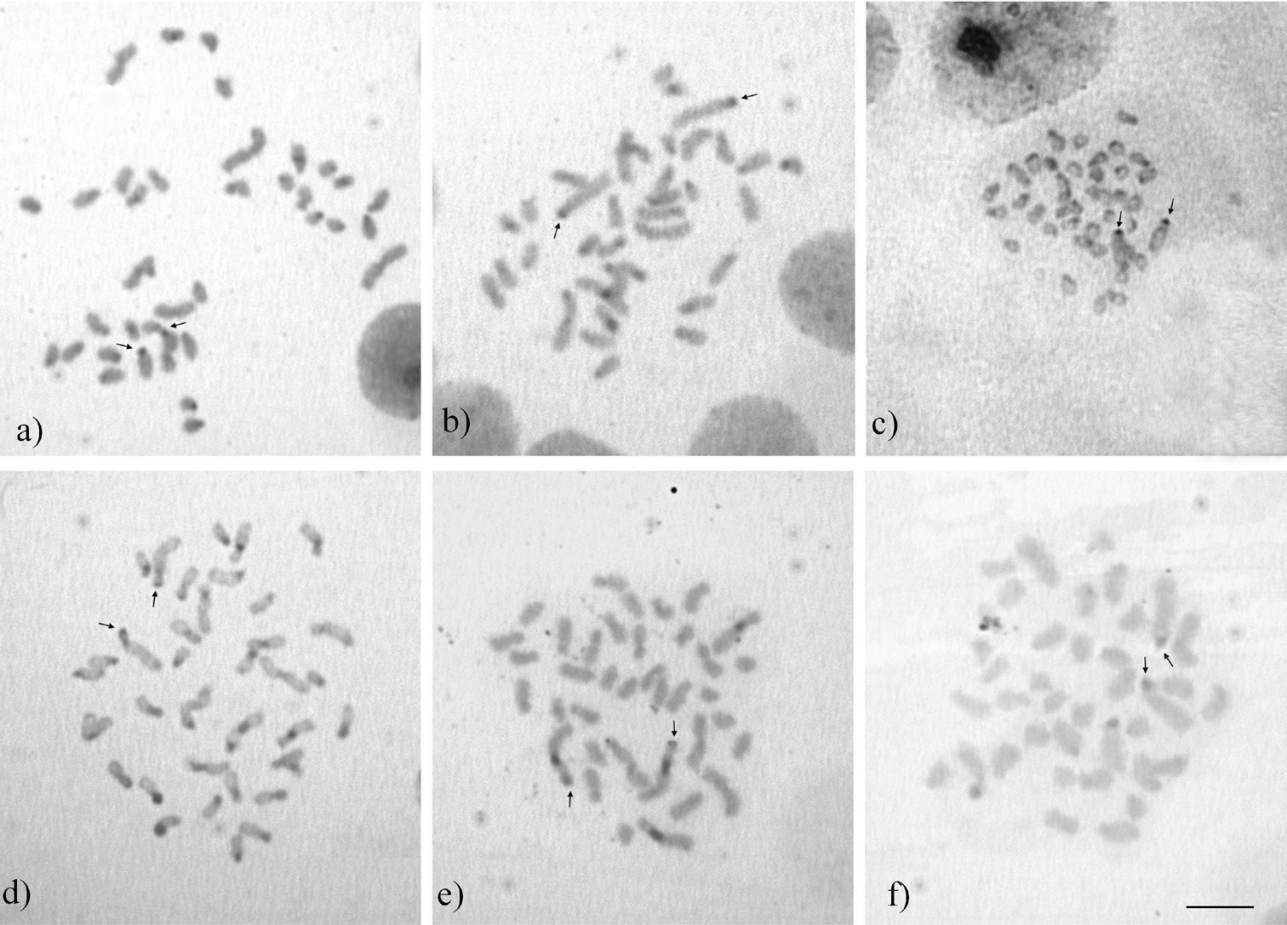
C-banded metaphases of *Acromyrmex* species. **a** *Acromyrmex balzani* **b** *Acromyrmex coronatus* **c** *Acromyrmex disciger* **d** *Acromyrmex rugosus* **e** *Acromyrmex niger* **f** *Acromyrmex echinatior*. Arrows indicate C-bands in the largest subtelocentric chromosome pair. Bar = 5 µm.

The C-banding results of *Acromyrmex balzani*, *Acromyrmex coronatus*, *Acromyrmex disciger* (Mayr, 1887) and *Acromyrmex rugosus* (Smith, 1858) indicated bands in some chromosomes: in the short arms of the submetacentric and subtelocentric and also in the centromeric regions of the metacentric chromosomes (Fig. 2). The largest subtelocentric pair (denominated as ST1) showed bands in all the species. *Acromyrmex disciger* (Fig. 2c), *Acromyrmex rugosus* (Fig. 2d) and *Acromyrmex coronatus* (Fig. 2b) had bands in the telomeric region of the short arm. In *Acromyrmex balzani* (Fig. 2a) and *Acromyrmex echinatior* (Fig. 2f) karyotypes, the heterochromatic bands were observed in the short arm of ST1 pair. *Acromyrmex niger* (Smith, 1858) (Fig. 2e) showed bands in the telomeric region of the short arm of the ST1 pair and also in the long arm. Moreover, *Acromyrmex rugosus* and *Acromyrmex niger* had additional bands in the pericentromeric region and in the long arm region of the second subtelocentric pair, respectively.

The short arms of the ST1 pair revealed differences in the banding patterns among the species with fluorochrome CMA_3_. *Acromyrmex disciger* (Fig. 3c) and *Acromyrmex coronatus* (Fig. 3b) in the telomeric regions. *Acromyrmex balzani* (Fig; 3a) in the short arms; and *Acromyrmex echinatior* in the interstitial region (Fig. 3f). However, *Acromyrmex niger* (Fig. 3e) and *Acromyrmex rugosus* (Fig. 3d), besides the telomeric regions of the ST1 pair, also showed bands in additional chromosomes. *Acromyrmex niger* (Fig. 3e) had additional bands in the long arm of the ST1 pair and in the long arm of the second larger subtelocentric pair. *Acromyrmex rugosus* (Fig. 3d) showed small bands in the telomeric regions of ST1 and in at least three other chromosomes.

**Figure 3.** Metaphases of *Acromyrmex* species stained with CMA_3_ and DAPI, respectively. **a** *Acromyrmex balzani* **b** *Acromyrmex coronatus* **c** *Acromyrmex disciger* **d** *Acromyrmex rugosus* **e** *Acromyrmex niger* **f** *Acromyrmex echinatior*. Arrows indicate CMA_3_-positive bands in the largest subtelocentric pair. Arrowheads indicate additional CMA_3_-positive bands in *Acromyrmex niger* and *Acromyrmex rugosus*. Bar = 5 µm.

**Figure 4.**
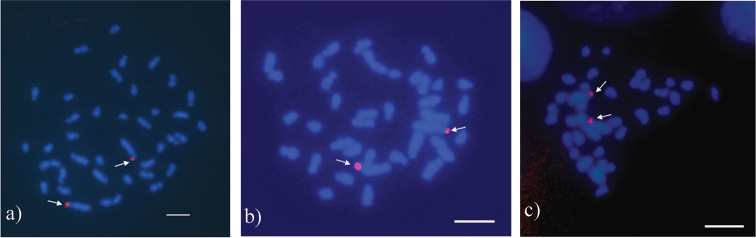
Metaphases of *Acromyrmex* species. FISH with 45S rDNA probe. **a** *Acromyrmex disciger* **b** *Acromyrmex coronatus* **c** *Acromyrmex niger*. Arrows indicate hybridization signals in the subtelocentric pair. Bar = 5 µm.

DAPI-positive bright bands which could correspond to AT-rich regions were not revealed (Fig. 3a–f). Instead, DAPI-negative regions which co-localized with CMA_3_-positive bands were visualized. FISH with the 45S rDNA probe visualized telomeric bands in ST1 pairs of *Acromyrmex coronatus*, *Acromyrmex disciger* and *Acromyrmex niger* (Fig. 4).

## Discussion

Karyotypes of *Acromyrmex* species observed in this study can be distinguished only on the basis of chromosomal measurements. Differential heterochromatin growth is therefore responsible for small but robust differences in chromosomal morphology, and these differences could not be observed using classification proposed by Imai (1991). This classification is based on heterochromatin distribution, and it does not reflect differences in the chromosome size among similar karyotypes. However, some chromosomes had the chromosome arm ratio (r) within the limits of the classification (submetacentric or subtelocentric). In the ants *Acromyrmex coronatus* and *Acromyrmex niger* the chromosomes were classified according to the greater ratio (r), i.e., as subtelocentrics.

The largest metacentric pair of *Acromyrmex balzani* strongly differs in size from that of other species probably due to complex chromosomal rearrangements that need to be further investigated. This can be explained by the fact that *Acromyrmex balzani* forms a separate clade together with *Acromyrmex landolti* according to the molecular phylogeny presented by Cristiano et al. (2013).

The six studied species showed heterochromatic segments on the short arms of ST1 pair. It was observed that these GC-rich heterochromatic regions correspond to NOR which is, in turn, confirmed by FISH with the 45S rDNA probe in the chromosomes of *Acromyrmex coronatus*, *Acromyrmex disciger* and *Acromyrmex niger*. In the latter species, this technique revealed a single NOR, although additional multiple CMA_3_-positive bands also were observed. This means that these additional bands are not related to the ribosomal genes. NORs are generally GC-rich and CMA_3_-positive in different organisms (Reed and Phillips 1995). However, CMA_3_-positive regions are not always rDNA clusters (Sumner 1990), as was observed in *Acromyrmex niger*. Multiple CMA_3_-positive bands and a single NOR revealed by FISH were observed in the fungus-growing ant *Mycocepurus goeldii* (Forel, 1893) (Barros et al. 2010, 2012); however, ribosomal gene mapping studies of Formicidae of the Neotropical region using FISH are scarce.

The nonspecific banding pattern of DAPI staining revealed in the present work is similar to those observed for other fungus-growing ants such as *Mycocepurus goeldii* (Barros et al. 2010), *Acromyrmex striatus* (Cristiano et al. 2013), *Trachymyrmex fuscus* Emery, 1934 (Barros et al. 2014) and *Atta* species (Barros et al. 2014, 2015).

Up to now, 12 *Acromyrmex* species (plus the only subspecies) are cytogenetically studied. All of them show 2n = 38, including both subgenera *Acromyrmex* and *Moellerius* (Fadini and Pompolo 1996, Goñi et al. 1983). However, *Acromyrmex striatus* with 2n = 22 differs from other already known species (Cristiano et al. 2013). The latter chromosome number is also characteristic of all *Atta* species. Both *Acromyrmex* and *Atta* are considered the most derived genera of fungus-growing ants (Schultz and Brady 2008, Mehdiabadi and Schultz 2009). Since differences in chromosomal morphology and banding patterns can be observed within *Acromyrmex*, it differs in this respect from *Atta*.

Patterns of heterochromatin distribution on short arms of some submetacentric and subtelocentric chromosomes of *Acromyrmex* species suggest that centric fissions which contributed to the origin of the derived karyotype with 2n = 38, probably occurred in the karyotype of the most recent common ancestor of this group. Moreover, recent molecular phylogenetic reconstruction by Cristiano et al. (2013) also suggests that *Acromyrmex striatus* is a sister group to the remaining leafcutter ants. The above-mentioned fissions were followed by heterochromatin growth which played an important role in maintaining telomeric stability according to the minimum interaction theory proposed by Imai et al. (1994). Differential heterochromatin growth in *Acromyrmex* is responsible for interspecific variation in the size of heterochromatic blocks. However, different species of this genus retain the same chromosome number (2n = 38), except for *Acromyrmex striatus* with 2n = 22 (Cristiano et al. 2013).

Besides data on the chromosome numbers, multiple GC-rich segments were observed in the fungus-growing ants *Mycocepurus goeldii* (Barros et al. 2010), *Sericomyrmex* sp. (Barros et al. unpublished data), *Trachymyrmex fuscus* (Barros et al. 2014b) and *Acromyrmex striatus* (Cristiano et al. 2013). Nevertheless, single CMA_3_-positive bands were found in the short arms of ST1 pairs in all *Acromyrmex* spp. and in the fourth chromosome pair of *Atta* species. Multiple GC-rich segments observed in *Acromyrmex niger* do not represent NORs, and therefore are probably derived.

Cytogenetic data permitted the differentiation among four of the six *Acromyrmex* species studied. *Acromyrmex balzani*, included in the *Moellerius* subgenus, showed the largest metacentric chromosome pair with lower size compared with the other species. *Acromyrmex echinatior*, besides the higher quantity of acrocentric chromosomes, also had interstitial bands in the ST1 pair for the fluorochrome CMA_3_, differing from the other species, which suggest the possibility of inversion. *Acromyrmex niger* showed multiple CMA_3_-positive bands: in the telomeric regions of the short arms of the ST1 pair, in the pericentromeric regions of the long arm of the ST1 pair and in the second largest subtelocentric pair. *Acromyrmex rugosus* had a greater proportion of metacentric chromosomes compared with the other *Acromyrmex* and also showed small bands in the telomeric regions of at least three other chromosomes. *Acromyrmex coronatus* and *Acromyrmex disciger* could only be cytogenetically differentiated from the other species by slight differences in the morphology that are probably due to the differential growth of heterochromatin on the short arms of the chromosomes.

Five of the six *Acromyrmex* species studied in this paper were collected in a particular area in the South East of South America. However, *Acromyrmex echinatior* was collected in Central America, which is more than 5,000 km from the main study area. Moreover, another three species analyzed by Goñi et al. (1983) were collected in Uruguay, expanding the knowledge of South American *Acromyrmex*. The chromosome number is uniform among different species of *Acromyrmex* (sensu stricto), although chromosome morphology and banding patterns of these ants allow the identification of some species via their karyotypes. However, karyotype structure of *Acromyrmex striatus* suggests that it belongs to a different lineage and therefore, according to sequences obtained from nuclear genes, this species does not belong to the “true” *Acromyrmex* lineage but to the sister group to the remaining leafcutter ants (Cristiano et al. 2013). A new genus therefore could be erected due to the karyotypic features of *Acromyrmex* which are further supported by combining the already published cytogenetic and molecular data (Cristiano et al. 2013) together with the additional karyological information. In this case, cytogenetics shows its importance as an additional tool in integrative taxonomy.

Conserved chromosome numbers were found in certain ant genera, as in *Pogonomyrmex* Mayr, 1868 in which 13 of 15 studied species had the same chromosome number. The two other species were transferred to another subgenus *Ephebomyrmex* Wheeler, 1902 (Taber et al. 1988). Camponotus (Myrmothrix) spp. also presented uniform chromosome number (Mariano et al. 2003), as did the other members of the genera *Pheidole* Westwood, 1839, *Lasius* Fabricius, 1804 and *Iridomyrmex* Mayr, 1862 (reviewed in Lorite and Palomeque 2010). Other animal groups, such as most birds and different genera of insects of the order Lepidoptera also demonstrated conservatism in respect to the chromosome number (White 1973), as did some bee genera, such as *Melipona* Illiger, 1806 (reviewed in Rocha et al. 2007) and *Partamona* Schwarz, 1939 (reviewed in Martins et al. 2009).

Our data confirmed uniformity of the chromosome number (2n = 38) in the studied *Acromyrmex* species. However, chromosomal rearrangements such as heterochromatin growth are likely to be responsible for karyotypic differentiation in this ant group. Location of rDNA clusters of other leafcutter ants (especially *Acromyrmex striatus*) also needs to be determined using molecular cytogenetic techniques (FISH). Moreover, cytogenetic studies of other members of fungus-growing ants, e.g. of the genus *Trachymyrmex* Forel, 1893 which represents the sister group to leafcutter ants, will be important for better understanding of chromosomal evolution of this group and Neotropical Formicidae in general.
